# Supplementary material for: Randomized control trial of moderate dose vitamin D alters microbiota stability and metabolite networks in healthy adults
Source: Microbiol Spectr. 2024 Aug 27;12(10):e00083-24. doi: 10.1128/spectrum.00083-24 (PMC11448053; doi:10.1128/spectrum.00083-24)
Supplement: Supplemental figures and tables — Fig. S1-S11; Tables S1-S4. [file spectrum.00083-24-s0001.pdf]

SUPPLEMENTARY MATERIALS

Title: Randomized control trial of moderate dose vitamin D alters microbiota stability and metabolite networks in healthy adults.

Authors: Madhur Wyatt [1], Ankan Choudhury [2], Ella Von Dohlen[2], Jeffery L. Heileson[1,3], Jeffrey S. Forsse[1,4], Sumudu Rajakaruna[5,6], Manja Zec[5,6], Malak M. Tfaily[5,6], and Leigh Greathouse [2,4*]

Author Affiliations:

[1] Human Health Performance and Recreation, Robbins College of Health and Human Sciences, Baylor University TX, USA
[2] Human Science and Design, Robbins College of Health and Human Sciences, Baylor University, TX, USA
[3] Nutrition Services Division, Walter Reed National Military Medical Center, MD, USA
[4] Department of Biology, Baylor University, TX, USA
[5] Department of Environmental Science, University of Arizona, 1177 E 4th St., AZ 85721, USA
[6] BIO5 Institute, The University of Arizona, 1657 E Helen St., Tucson, AZ 85719, USA
* Correspondence: leigh_greathouse@baylor.edu; Tel.: +1-254-710-7619

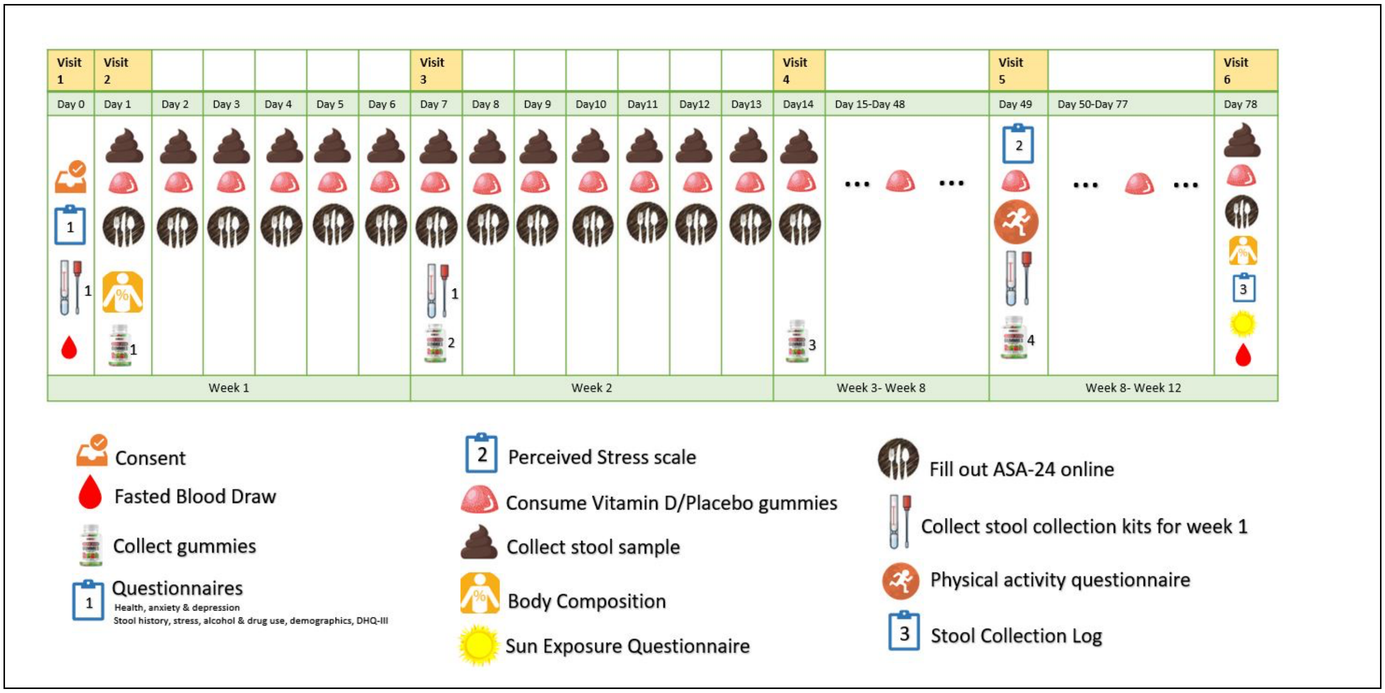

Figure S1. VDMT Experiment Design: 12-week randomized placebo control trial

Sprintec	Adderall XR
Montelukast	Lexapro
Vyvance	Spironolacton
Lunesta	Exemestane
Sertraline	Lamictal
Symbicort	Adderall
Hydroxyzine	Singulair
Accutane	Trazadone

Table S1. List of drugs taken by all participants during the study

**Vitamin D**	**Placebo**
Vitamin D (Cholecalciferol)	No Vitamin D
Organic tapioca syrup	Organic rice syrup
Organic cane sugar	Organic cane sugar
Pectin	Pectin
Citric acid	Citric acid
Fumeric acid	Ascorbic acid
Natural flavor<br>Fruit and vegetable juice (color)	Natural flavors colored with organic concentrate (apple, carrot, pumpkin, bluecurrent)
Sodium citrate dihydrate	Organic sunflower oil
Purified water	Organic carnauba wax
Gluten-free	Gluten-free
Contains milk derivatives	Vegan
Non-GMO	Non-GMO

Table S2. List of ingredients in vitamin D and placebo gummies provided to the participants during a 12-week placebo-control trial.

Placebo			Treatment		
**Participant**	**Baseline**	**Post Intervention**	**Participant**	**Baseline**	**Post Intervention**
6	39.644	36.308	1	30.922	70.151
8	25.662	34.682	2	26.295	52.475
9	62.101	42.514	3	20.858	75.695
10	49.757	44.869	4	28.874	61.912
13	49.12	43.696	5	30.08	49.058
14	58.835	62.484	7	52.221	74.913
23	20.472	31.61	11	40.208	72.351
24	31.044	28.617	12	43.702	45.404
26	27.671	24.367	15	60.006	142.885
29	29.608	38.601	16	47.81	51.717
30	47.626	38.959	17	44.722	84.751
31	40.311	43.814	18	41.358	64.77
32	30.12	30.1	19	23.574	73.446
33	13.711	17.341	20	34.9	52.617
34	43.87	48.481	21	Withdrew for medical reasons	
36	53.423	32.157	22	15.926	46.748
37	35.126	35.77	25	52.463	85.229
38	21.4	28.394	27	51.256	63.586
40	Unable to draw the blood sample		28	76.895	152.774
41	51.588	27.815	35	21.522	53.879
42	22.578	29.38	43	49.183	77.879
44	41.784	38.565			

Table S3. Baseline and post-intervention serum 25(OH)D levels among placebo and treatment groups (measured in ng/mL).

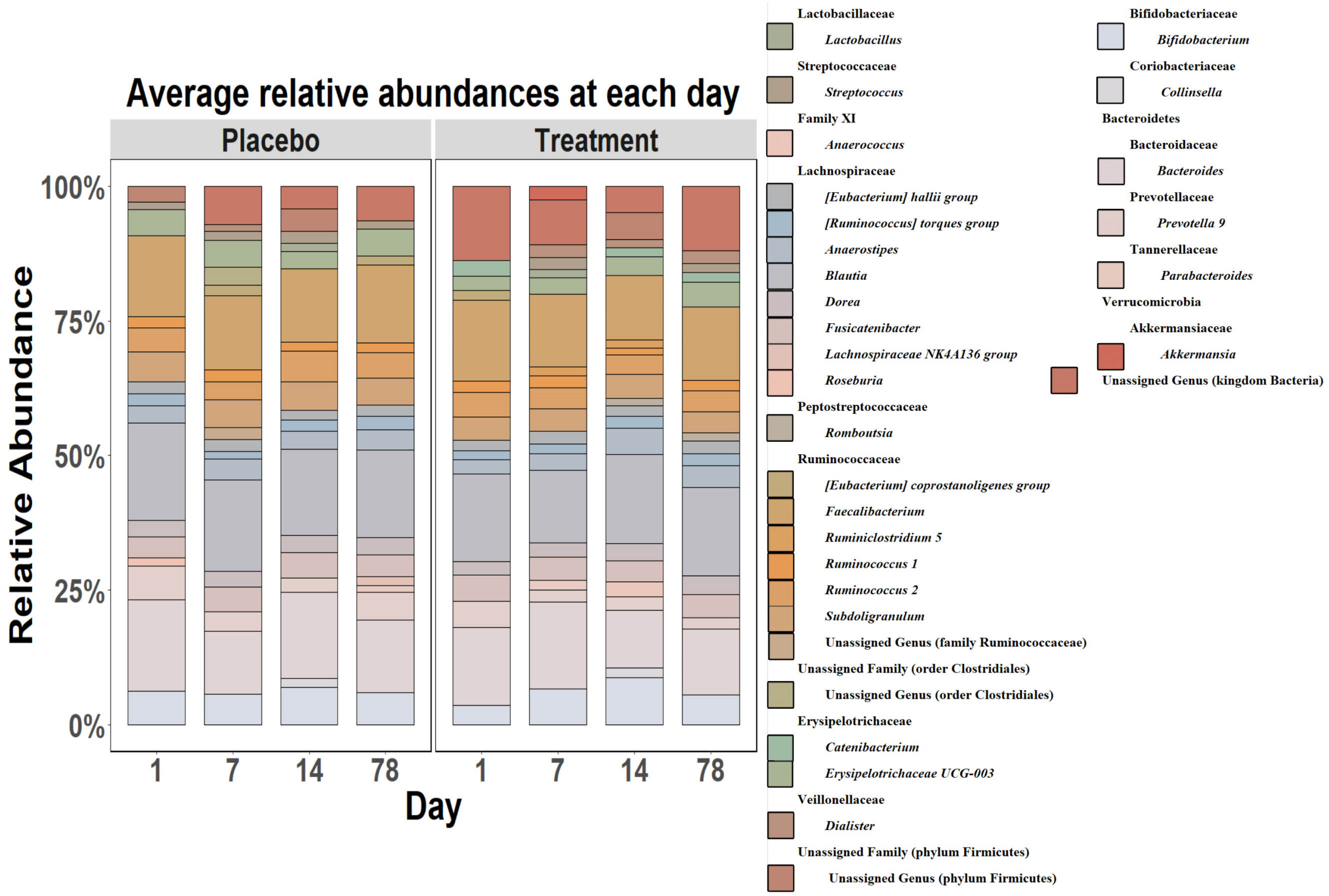

Figure S2. Changes in relative abundances of the most abundant genera of gut microbiota across the 4 days of sampling in both placebo and treatment groups.

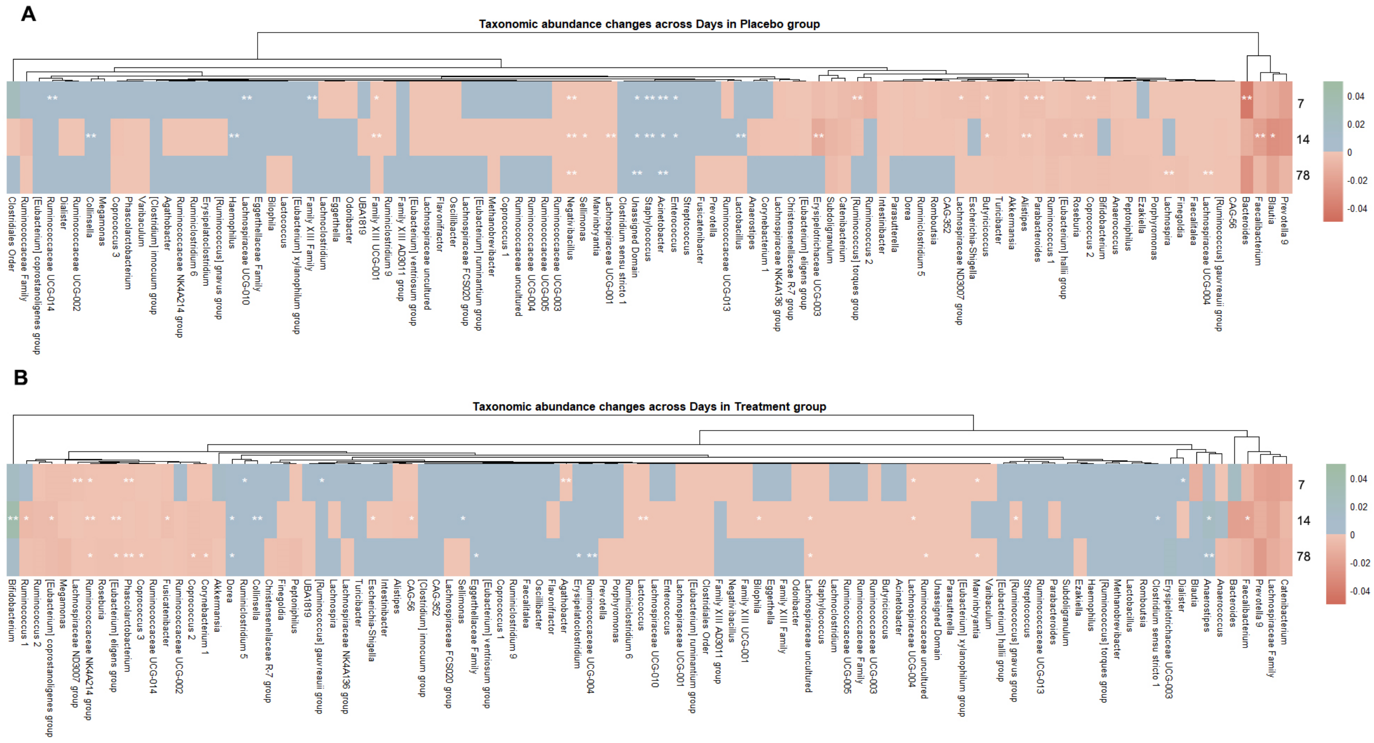

Figure S3. Overall microbial taxonomic abundance at the genus level by time points and groups (Placebo and treatment). The three rows represent taxonomic changes from days 1-7, 7-14, and 14-78 in order. Blue represents a decrease in microbial population while yellow represents an increase in microbial population (*;*p*<.05, **;*p*<.01). A. Microbial taxonomic abundance at the genus level by timepoints in the treatment group. B. Microbial taxonomic abundance at the genus level by time points in the placebo group.

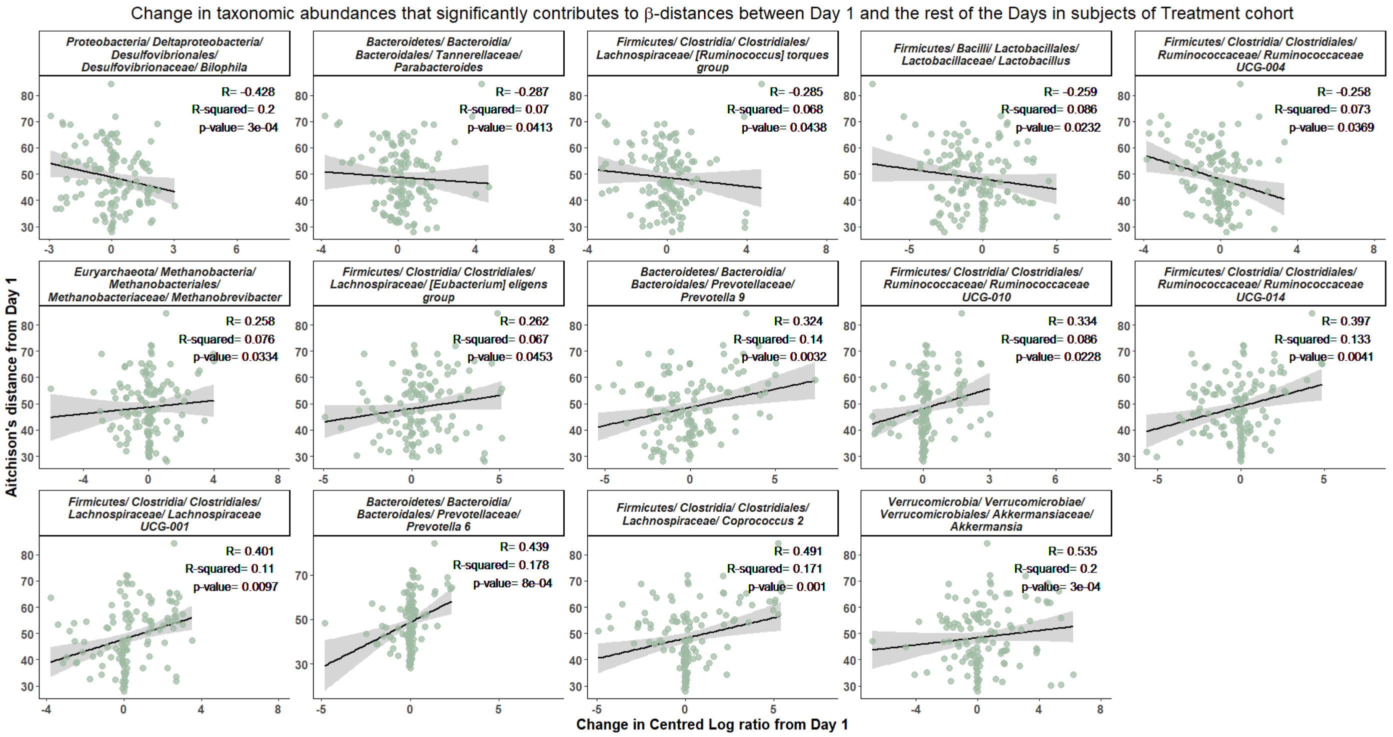

Figure S4. Taxonomic abundance change that significantly correlated (Pearson's, FDR adjusted <0.05) with the beta distance (Aitchison's) of the Treatment subjects in subsequent days (7, 14, and 78) from Day 1 of the experiment. A) The change in Centred log-ratio of the taxa from day 1 to the subsequent days correlated against the beta diversity distance from day 1, B) The ranking of different taxa based on their correlation against the beta diversity distance of samples from subsequent days from samples of day 1 for Treatment subjects.

Bacterial Taxon	VDMT (Treatment group)	Timepoint	CRC	Mechanism
*Anaerostipes***	Increasing	Day 7-14<br>Day 14-78	Low (in fecal[107] and tissues samples[98])	Butyrate Production
Bifidobacterium **	Increasing	Day 7-14	Low (in fecal sample[84])	Reduce Zonulin (increases gut permeability), reduces pro-inflammatory cytokines- TNF-α, IL-10, IL-12
*Lactococcus***	Decreasing	Day 7-14	High<br>(in cancer tissue compared to adjacent non-cancerous tissue[108])	Might be involved in carcinogenesis by decreasing number of natural killer (NK) cells[109]. No studies found analyzing stool sample studies for Lactococcus.
*Agathobacter***	Decreasing	Day 1-7	No information found in context of CRC. But, in healthy controls, Agathobacter was found to be higher in fecal samples[110]	Butyrate producer[111]
*Collinsella***	Increasing	Day 7-14		Alter gut permeability.<br>Collinsella has been associated with low fiber intake diets in individuals with obesity, nonalcoholic steatohepatitis, rheumatoid arthritis[112–114]. In a CRC vs healthy cohort, Collinsella was higher in healthy controls[110]
*Phascolarctobacterium***	Decreasing	Day 1-7<br>Day 7-78	Higher in fecal samples of individuals with CRC[102,115].	SCFA production, including acetate and propionate[116]
*Eubacterium eligens group***	Decreasing	Day 7-14<br>Day 14-78		Anti-inflammatory, IL-10 production, increased SCFA production[117,118]<br>Higher proportion of *Eubacterium eligens group* found in fecal samples of healthy adults compared to individuals with CRC[119–121]
*Ruminococcus NK4A214***	Decreasing	Day 7-14	Enriched in CRC fecal samples compared to healthy controls[122]	Microbes in genus *Ruminococci* has shown to degrade cellulose in the gut[123].
*Faecalibacterium**	Decreasing	Day 7-14	Reduced in intestinal lumen microbiota, mucosa-adherent microbiota of patients with CRC[124]	Anti-inflammatory effects by blocking NF- κB expression and IL-8 secretion[124].
*Dialister**	Increasing	Day 1-7	High in on-tumor and adjacent-tumor, and of patients with CRC[125]	Potential biomarker for CRC recurrence and patient prognosis[125]
*Clostridium sensu stricto 1**	Increasing	Day 7-14	Higher in CRC group compared to control healthy mice[126]	
*Ruminococcus 1**	Decreasing	Day 7-14	Depleted in fecal samples of patients with CRC compared to healthy controls[127]	Associated with insulin resistance, higher HbA1c, and inflammation[128]

*Bilophila wadsworthia**	Decreasing	Day 7-14	High abundance in colonic mucosa in patients with CRC[129].	Inflammation, gut permeability, bile acid and glucose dysmetabolism, hepatic steatosis, genotoxic hydrogen sulfide production[98,130,131].
*Eggerthellaceae family**	Increasing	Day 14-78	Enriched in on-tumor microbiota in patients with left-sided CRC[132]	Anti-inflammatory. This microbial family has been isolated from fecal samples from healthy adults as well.
Escherichia-Shigella	Decreasing	Day 7-14	Enriched in fecal microbiota of patients with preneoplastic lesions, including adenomas and hyperplastic polyps[133]. Enriched in fecal microbiota of patients with CRC with hyperlipoidemia and hypercholesteremia[134]	Genotoxin-producers, carcinogenic[133]

Table S4. Comparison of individual microbial taxonomic abundance in VDMT and their relationship to CRC-associated bacteria. All bacteria listed were seen to fluctuate in stool samples of treatment group with significance <.01 (**) and <.05 (*). Blank table cells represent limited knowledge in the field.

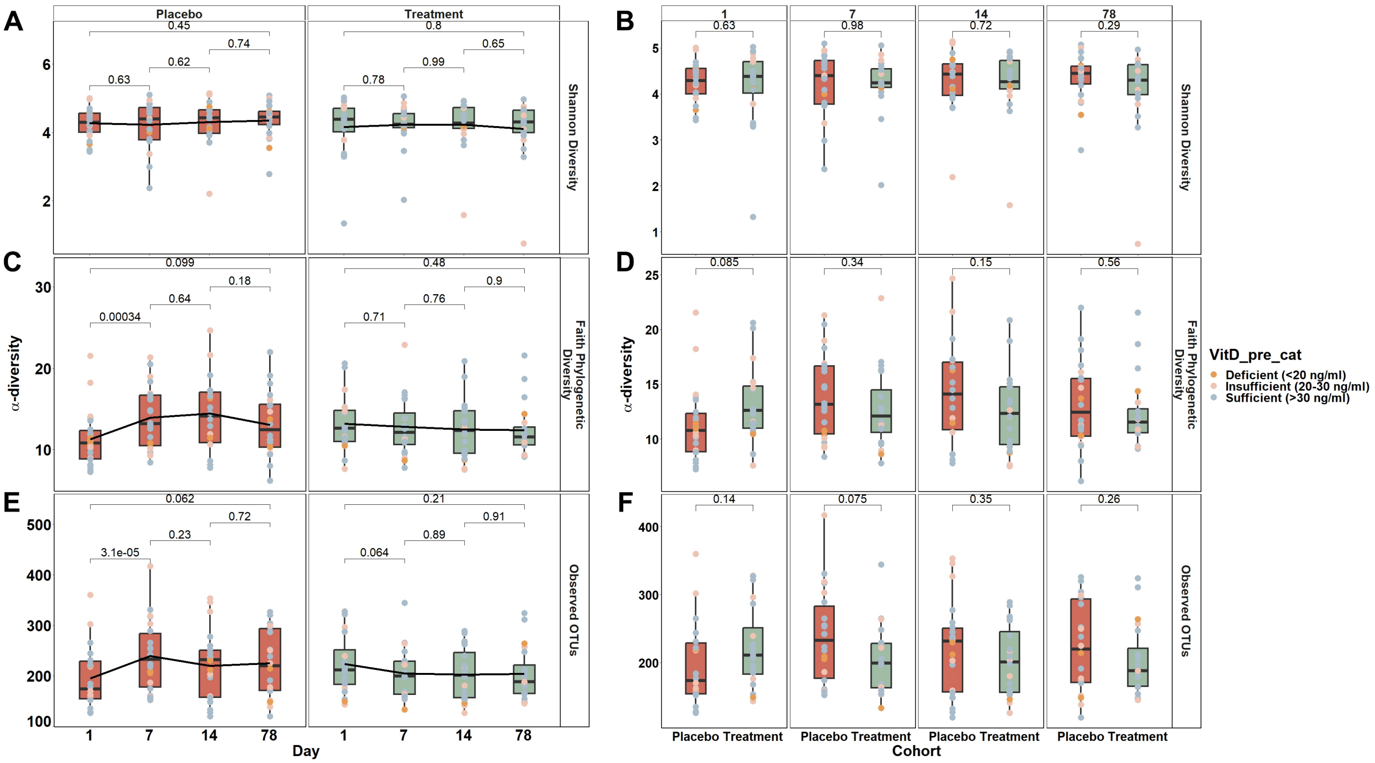

Figure S5. Comparison of α-diversity by timepoint between and within the treatment and placebo group. (A). α-diversity- Shannon Diversity Index. The comparison of microbial alpha diversity using Shannon Diversity Index by timepoints- Day 1, 7, 14, and 78 within the groups (left) and between groups (right). The different color dots in each graph represents the different serum 25(OH)D levels measured in ng/mL. Red dots represent participants with 25(OH)D deficiency (below 20 ng/mL), green dots represent

participants with 25(OH)D insufficiency (21-29 ng/mL), and blue dots represent participants with 25(OH)D sufficiency (above 30 ng/mL). (B). The comparison of microbial alpha diversity using Faith Phylogenetic Diversity by timepoints within the groups (left) and between groups (right). (C). The comparison of microbial alpha diversity using ASVs by timepoints within the groups (left) and between groups (right). (Statistical Test: Wilcoxon's Paired Test)

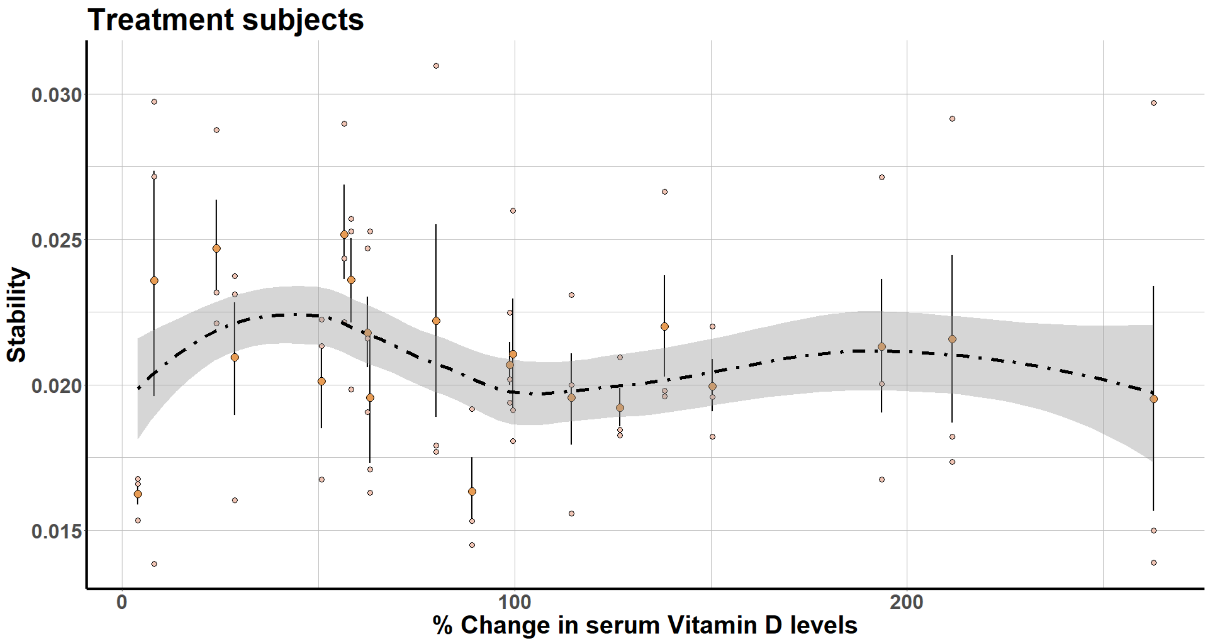

Figure S6. Range of stability (inverse Aitchison's distance between samples from consecutive timepoints) seen in every individual among the Treatment cohort plotted against the %change in their serum Vitamin D (ball and stick figures denote the mean and 1st standard deviation range for each subject respectively).

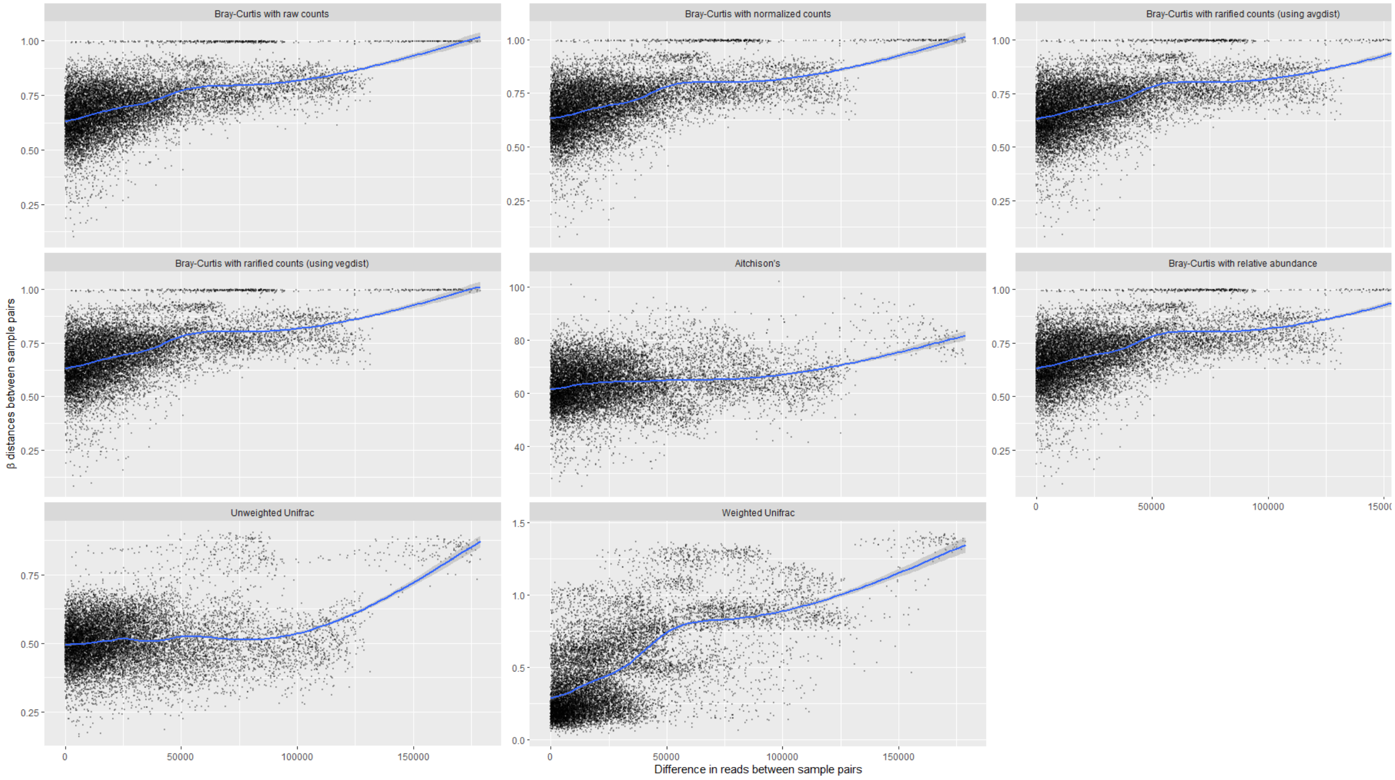

Figure S7. Comparison of commonly used distance matrices with number of reads between sample pairs to determine which of these matrices is the best fit for our VDMT microbiota data. Aichison's distance was identified as the most robust to differences in number of reads per sample and hence was chosen for analysis of our data.

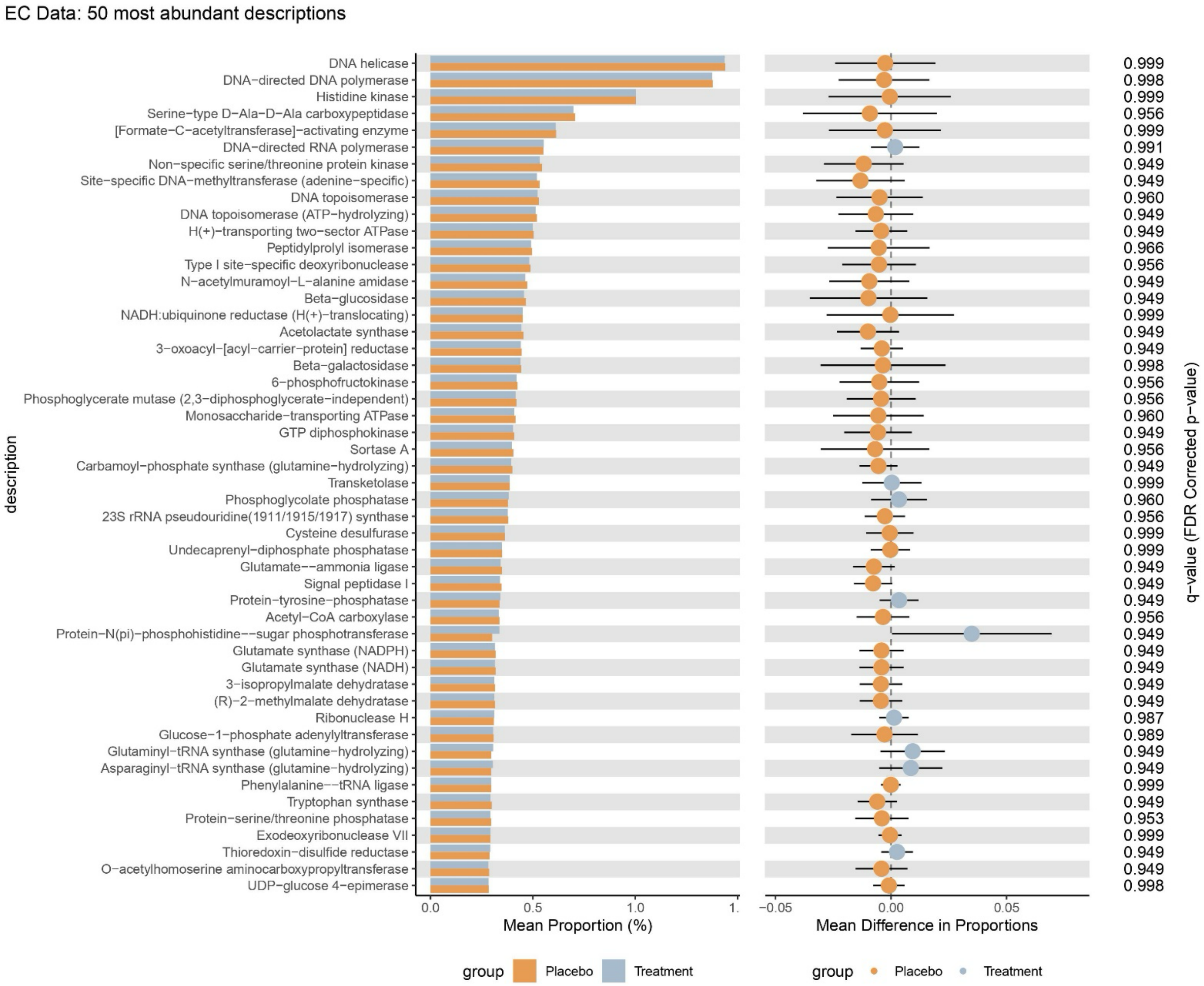

Figure S8. Inferred microbial pathway analysis between placebo and treatment groups. Mean proportion and difference in proportions of PICRUSt2 predicted Enzyme Commission numbers (EC numbers). Mean relative abundance of each output was calculated and a general linear model was used to calculate differential abundance between placebo and treatment groups with subject as a fixed effect. FDR corrected *P* values <0.1 were considered significant.

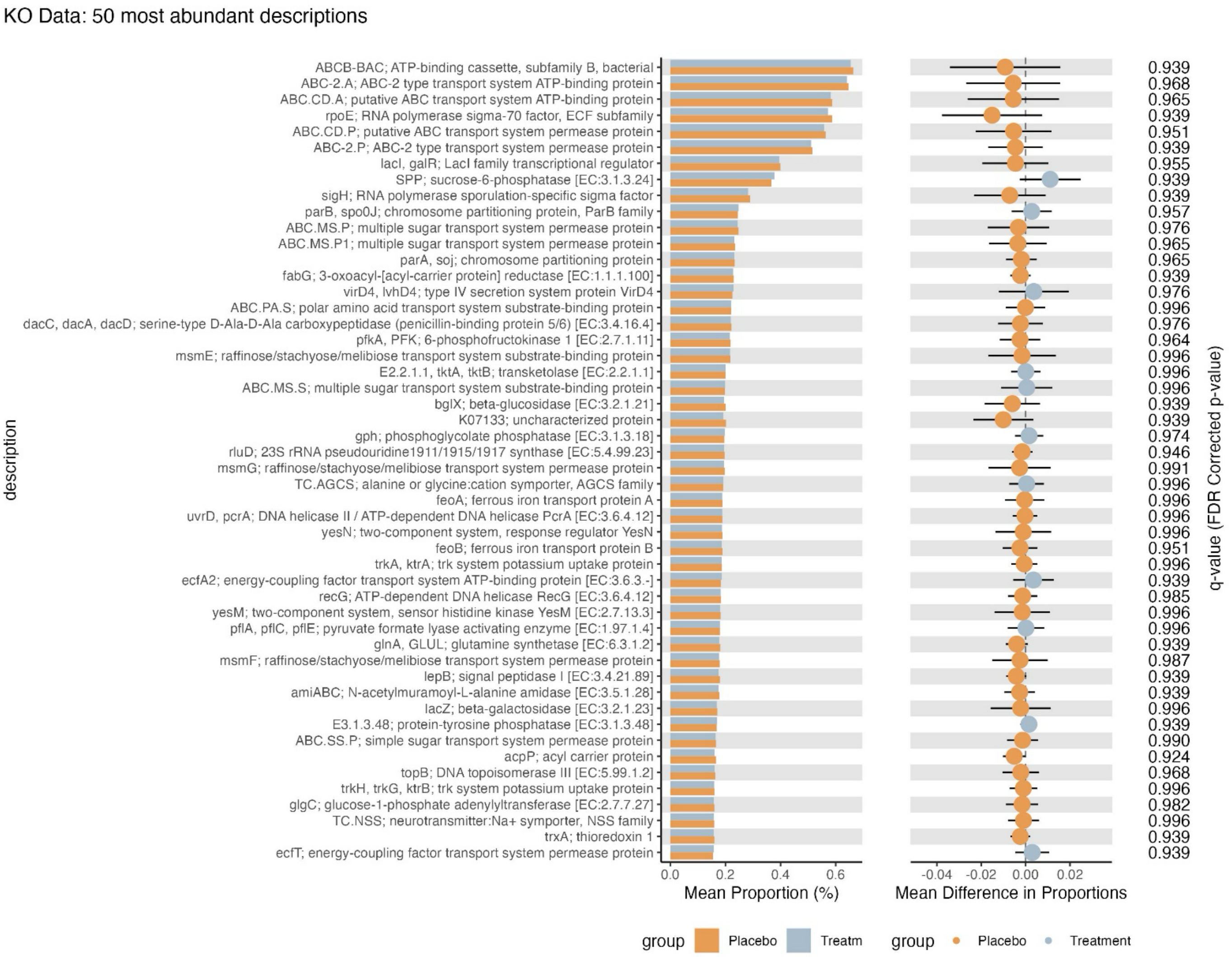

Figure S9. Inferred microbial pathway analysis between placebo and treatment groups. Mean proportion and difference in proportions of PICRUSt2 predicted KEGG orthologs (KO). Mean relative abundance of each output was calculated and a general linear model was used to calculate differential abundance between placebo and treatment groups with subject as a fixed effect. FDR corrected *P* values <0.1 were considered significant.

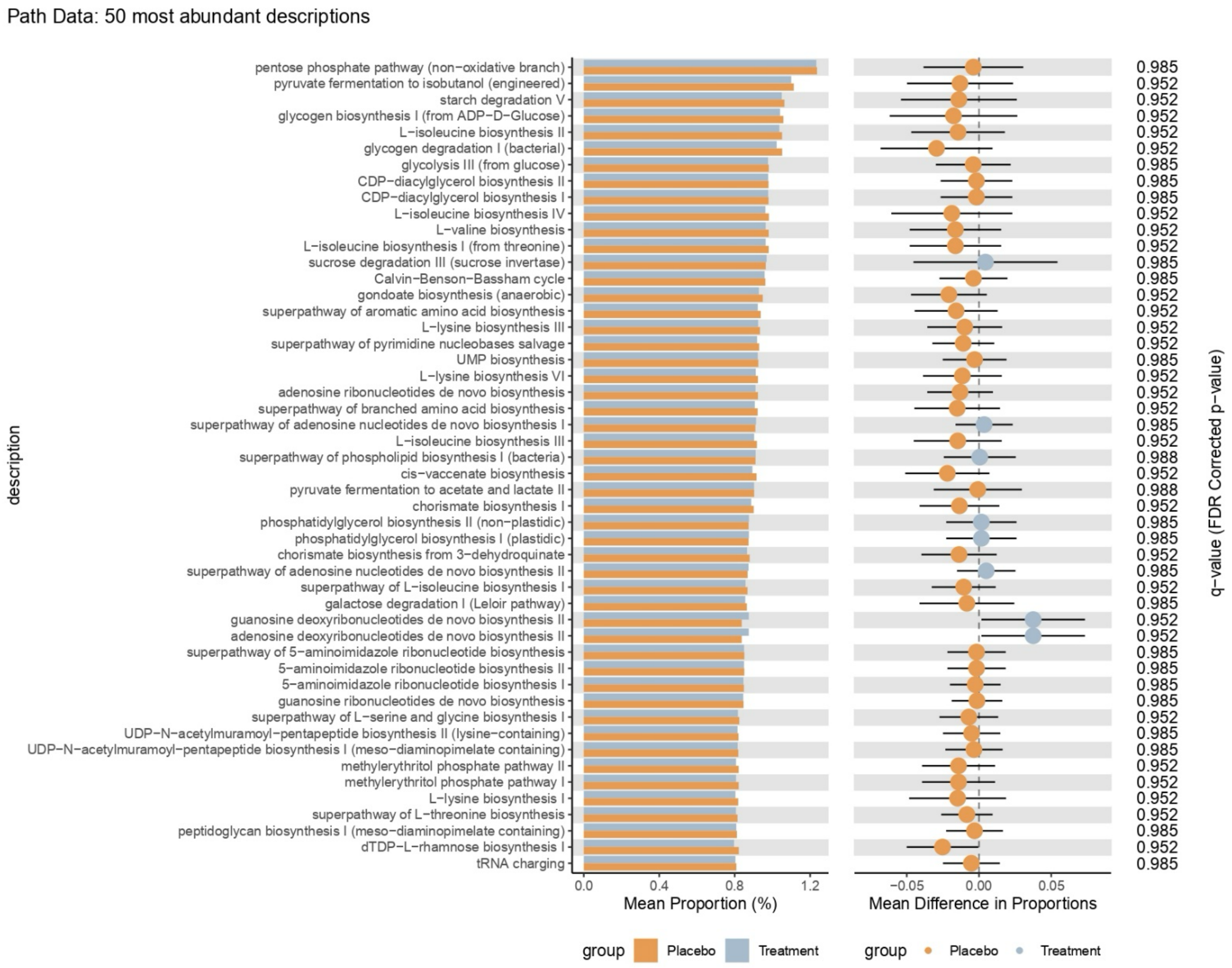

Figure S10. Inferred microbial pathway analysis between placebo and treatment groups. Mean proportion and difference in proportions of PICRUSt2 predicted metabolic pathways between placebo and treatment microbial datasets. Mean relative abundance of each output was calculated and a general linear model was used to calculate differential abundance between placebo and treatment groups with subject as a fixed effect. FDR corrected *P* values <0.1 were considered significant.

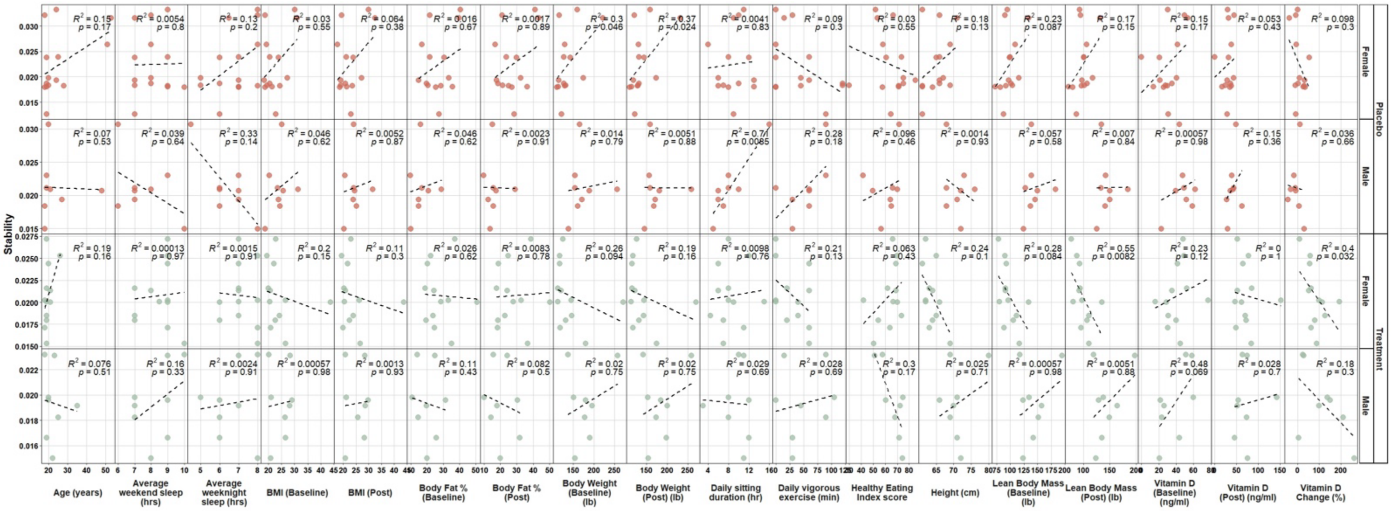
Stability
Female
Male
Placebo
Treatment
Age (years)
Average weekend sleep (hrs)
Average weeknight sleep (hrs)
BMI (Baseline)
BMI (Post)
Body Fat % (Baseline)
Body Fat % (Post)
Body Weight (Baseline) (lb)
Body Weight (Post) (lb)
Daily sitting duration (hr)
Daily vigorous exercise (min)
Healthy Eating Index score
Height (cm)
Lean Body Mass (Baseline) (lb)
Lean Body Mass (Post) (lb)
Vitamin D (Baseline) (ng/ml)
Vitamin D (Post) (ng/ml)
Vitamin D Change (%)

(A)

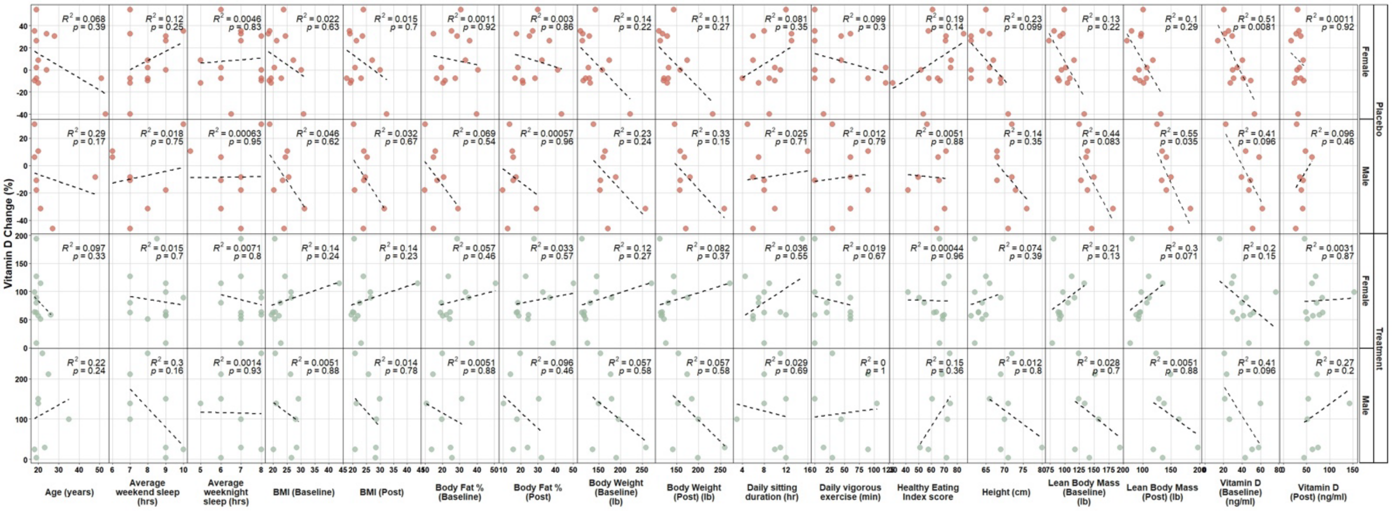
Vitamin D Change (%)
Female
Male
Placebo
Treatment
Age (years)
Average weekend sleep (hrs)
Average weeknight sleep (hrs)
BMI (Baseline)
BMI (Post)
Body Fat % (Baseline)
Body Fat % (Post)
Body Weight (Baseline) (lb)
Body Weight (Post) (lb)
Daily sitting duration (hr)
Daily vigorous exercise (min)
Healthy Eating Index score
Height (cm)
Lean Body Mass (Baseline) (lb)
Lean Body Mass (Post) (lb)
Vitamin D (Baseline) (ng/ml)
Vitamin D (Post) (ng/ml)

(B)

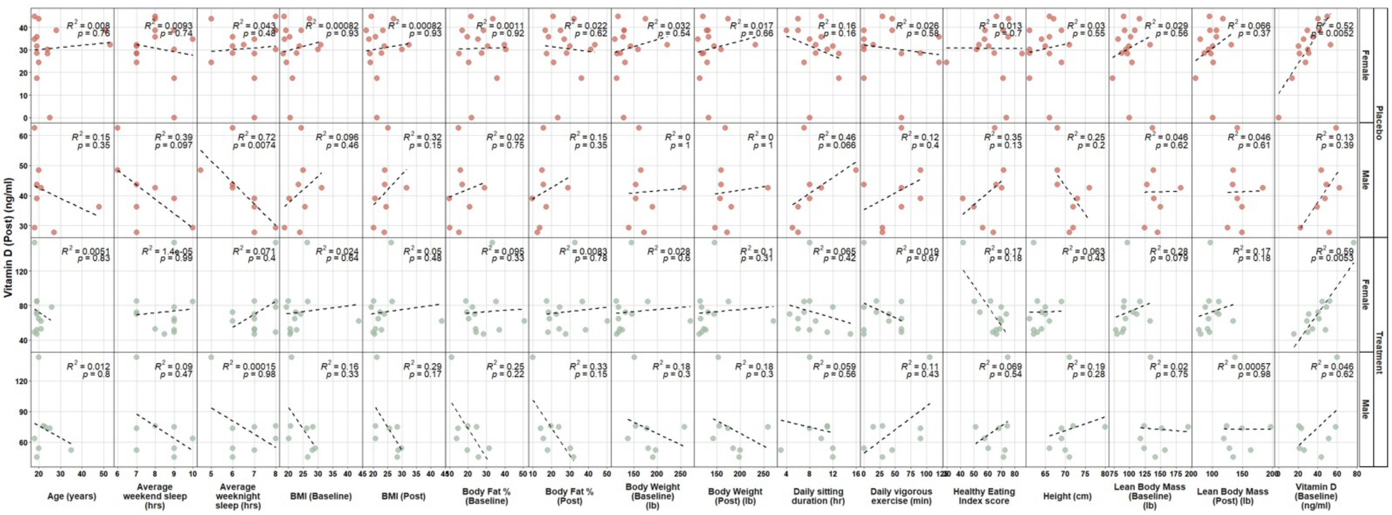
Vitamin D (Post) (ng/ml)
Female
Male
Placebo
Treatment
Age (years)
Average weekend sleep (hrs)
Average weeknight sleep (hrs)
BMI (Baseline)
BMI (Post)
Body Fat % (Baseline)
Body Fat % (Post)
Body Weight (Baseline) (lb)
Body Weight (Post) (lb)
Daily sitting duration (hr)
Daily vigorous exercise (min)
Healthy Eating Index score
Height (cm)
Lean Body Mass (Baseline) (lb)
Lean Body Mass (Post) (lb)
Vitamin D (Baseline) (ng/ml)

(C)

Figure S11. Linear Regression and correlation models (Spearman's) of the body composition variables, physical activity variables, sleeping behavior variables, dietary score, age, and Vitamin D (baseline, post-intervention, and % change) levels with Microbiome Stability (as described in Figure 3 (D)) (A), Vitamin D change % (B) and Vitamin D level-post intervention.
